# Neuralgic Amyotrophy Presenting with Multifocal Myonecrosis and Rhabdomyolysis

**DOI:** 10.7759/cureus.7382

**Published:** 2020-03-23

**Authors:** Michael R Goetsch, Jeffrey Shen, Jeffrey A Jones, Adeel Memon, Walter Chatham

**Affiliations:** 1 Medicine, Johns Hopkins Hospital, Baltimore, USA; 2 Internal Medicine, University of Alabama at Birmingham, Birmingham, USA; 3 Pathology, University of Alabama at Birmingham, Birmingham, USA; 4 Neurology, University of Alabama at Birmingham, Birmingham, USA; 5 Medicine, University of Alabama at Birmingham, Birmingham, USA

**Keywords:** myonecrosis, neuralgic amyotrophy, parsonage turner syndrome, brachial neuritis, neuropathy, rhabdomyolysis, brachial plexitis, brachial plexopathy, shoulder girdle

## Abstract

Neuralgic amyotrophy (NA), also known as Parsonage-Turner syndrome, is an idiopathic disorder characterized by rapid-onset unilateral upper extremity pain, paralysis, and sensory disturbance in the distribution of the brachial plexus. The etiology is unknown, and there is a multitude of alternative clinical presentations as well as secondary triggers, which make the diagnosis challenging. To date, there has been no report of NA presenting with frank myonecrosis. In this report, we document the first case of NA presenting with multifocal myonecrosis of the shoulder girdle muscles and rhabdomyolysis. This case posed a unique challenge in the diagnostic workup and management as many causes of myonecrosis present similarly to NA, and NA is a diagnosis of exclusion. Our patient underwent exhaustive testing and several trials of therapy before diagnosis could be made. Such evaluations are expensive and carry risks for patients. As such, it is important that physicians recognize this unique presentation of NA.

## Introduction

Neuralgic amyotrophy (NA) is an idiopathic diagnosis, previously thought to be quite rare but whose incidence in recent years has been estimated at up to 20-30 cases per 100,000 individuals [1]. While the pathophysiology is poorly understood, it is thought to involve an underlying genetic predisposition followed by an immune, autoimmune, mechanical, or infectious trigger [1-5]. The diagnosis is made clinically, and 96% of patients present with classical phenotypic features including severe, sudden onset, relentless, and asymmetric upper extremity pain with sensory and motor deficits in the distribution of the brachial plexus [1,2,6]. Patients commonly display winging of the scapula on the affected side(s), and pain frequently awakens patients from sleep [1,3]. NA is a diagnosis of exclusion that is primarily made clinically but supported by electrodiagnostic and imaging findings [1,2,5]. Electromyogram (EMG) and nerve conduction studies (NCS) show decreased sensory nerve action potentials, acute denervation, axonal damage, and/or reinnervation. Imaging studies mainly serve to exclude other causes, but MRI may show edema, thickening, and/or enhancement of involved muscles and nerves [1,2,5]. The distribution of involvement can be highly variable involving the nerves and muscles of the shoulder and trunk, and it can be difficult to diagnose as symptoms can be similar to other common orthopedic and neurologic injuries [3,6-8]. Our case was especially challenging given that in addition to the classic features, our patient had the atypical finding of myonecrosis that has not been previously described in the literature. This myonecrosis caused concern for an infectious etiology of our patient’s symptoms, delaying diagnosis and treatment.

## Case presentation

A 54-year-old Caucasian male was in his usual state of health when he developed mild numbness and tingling of his left hand. Within a few days, he awakened suddenly with severe pain, numbness, and paralysis of his entire left upper extremity (LUE). At the time of presentation, he denied any recent trauma or injury, vaccination, surgery, respiratory or gastrointestinal illness, fevers, chills, or sweats. He also denied any alcohol, tobacco, or illicit drug use. Review of systems was otherwise negative. He had a history of bilateral hip arthroplasty secondary to dysbaric osteonecrosis and numerous cervical spinal operations for repetitive mechanical trauma and barotrauma incurred during military service, but he retained full functional status without neurologic deficits. He had been retired for over 20 years at the time of presentation and had not performed any high altitude jumps or deep-sea dives in six years.

At the initial presentation, he was afebrile and his vitals were within normal limits. Detailed physical examination showed winging of the scapula on the left side and weakness in all muscle groups (1/5 strength) of the LUE, which could not be completely accounted for by pain. On further examination of the LUE, he had decreased tone, mild diffuse atrophy, and fasciculations. Light touch, pinprick, and temperature sensation were absent, and he displayed diffuse tenderness to palpation, pain with both passive and active range of motion, and areflexia. Examination of his right upper extremity showed 4/5 strength throughout but was otherwise without abnormalities. Laboratory evaluation revealed evidence of muscle injury (creatine kinase: 8,791 units/L) and acute kidney injury. Creatinine was 3.2 mg/dL from a baseline of 0.7 mg/dL. He also had elevated serum inflammatory markers (erythrocyte sedimentation rate: 33 mm/h; C-reactive protein: 15 mg/L). The remaining presenting lab values were within normal limits. Magnetic resonance imaging (MRI) of his shoulders showed diffuse multifocal edema of the shoulder girdle muscles bilaterally, diffuse inflammation of the brachial plexus bilaterally, and frank areas of necrosis in the right supraspinatus and left subscapularis muscles concerning for purulent infection. Left shoulder joint aspiration was performed, and gram stain and culture of the aspirate were negative. Tuberculin skin testing was non-reactive. An ultrasound-guided core biopsy of the necrotic lesion in his left subscapularis muscle was obtained, which demonstrated fragments of skeletal muscle with focal necrosis, chronic inflammation, and degenerative changes, but was otherwise without specific diagnostic findings. Culture and gram stain for this biopsy also returned negative for evidence of infection.

Given the severity of his clinical symptoms, imaging findings of multifocal edema and myonecrosis, and potential exposures to atypical organisms during his military career, concern for infectious etiology remained high. The presumed diagnosis was necrotizing pyomyositis, and he was started on an empiric course of vancomycin and cefepime to prevent a possible rapid destruction of the shoulder girdle. He was discharged home with a peripherally inserted central catheter (placed on the right side) with plans to complete a six-week course of antibiotics. His severe pain was treated with gabapentin, tizanidine, duloxetine, acetaminophen, and opioid analgesics with minimal relief. After completion of this course of antibiotics, he returned to the clinic with persistent symptoms and minimal improvement and was readmitted for further evaluation.

Bilateral upper extremity EMG and NCS were performed at two months after symptom onset and revealed acute-on-chronic severe left brachial plexopathy involving all levels, and chronic right brachial plexopathy (Tables 1-4).

**Table 1:** Nerve Conduction Study Anti-Sensory Summary NR, not recorded; ms, milliseconds; µV, microvolts; m/s, meters per second *Abnormal values

Stimulation Site	NR	Onset (ms)	Peak (ms)	Normal Peak (ms)	Amplitude (µV)	Normal Amplitude (µV)	Site 1	Site 2	Delta-0 (ms)	Distance (cm)	Velocity (m/s)	Normal Velocity (m/s)
Left Lateral Antebrachial Cutaneous Anti-Sensory (Lateral Forearm)												
Lateral Biceps	NR	-	-	<2.8	-	>12	Lateral Biceps	Lateral Forearm	-	12.0	-	>50
Right Lateral Antebrachial Cutaneous Anti-Sensory (Lateral Forearm)												
Lateral Biceps	NR	-	-	<2.8	-	>12	Lateral Biceps	Lateral Forearm	-	12.0	-	>50
Left Medial Antebrachial Cutaneous Anti-Sensory (Medial Forearm)												
Elbow		4.1	4.8*	<2.8	8.4*	>9	Elbow	Medial Forearm	4.1	12.0	29*	>50
Right Medial Antebrachial Cutaneous Anti-Sensory (Medial Forearm)												
Elbow		1.7	2.3	<2.8	11.1	>9	Elbow	Medial Forearm	1.7	11.0	65	>50
Left Median Anti-Sensory (2nd Digit\Wrist)												
Wrist	NR	-	-	<3.6	-	>15	Wrist	2nd Digit\Wrist	-	13.0	-	>50
Right Median Anti-Sensory (2nd Digit\Wrist)												
Wrist		2.5	3.3	<3.6	18.2	>15	Wrist	2nd Digit\Wrist	2.5	13.0	52	>50
Left Radial Anti-Sensory (Base 1st Digit)												
Wrist		2.3	2.9	<3.1	4.4*	>15	Wrist	Base 1st Digit	2.3	12.0	52	>50
Right Radial Anti-Sensory (Base 1st Digit)												
Wrist		1.9	2.7	<3.1	27.7	>15	Wrist	Base 1st Digit	1.9	12.0	63	>50
Left Ulnar Anti-Sensory (5th Digit)												
Wrist		4.3	4.8*	<3.5	7.8*	>12	Wrist	5th Digit	4.3	11.0	26*	>50
Right Ulnar Anti-Sensory (5th Digit)												
Wrist		2.2	2.8	<3.5	12.3	>12	Wrist	5th Digit	2.2	11.0	50	>50

**Table 2:** Nerve Conduction Study Motor Summary ms, milliseconds; mVms, millivolts·milliseconds; mV, millivolts; cm, centimeters; m/s, meters per second; A elbow, above the elbow; B elbow, below the elbow *Abnormal values

Stimulation Site	Onset (ms)	Negative Duration (ms)	Negative Area Under the Curve (mVms)	Normal Amplitude (mV)	Amplitude (mV)	Site 1	Site 2	Delta-0 (ms)	Distance (cm)	Velocity (m/s)	Normal Velocity (m/s)
Left Median Motor (Abductor Pollicis Brevis)											
Wrist	3.6*	5.55	10.47	>7	3.8*	Wrist	Wrist	0.0	5.0	-	>50
Elbow	8.9	6.88	8.97		3.0	Wrist	Elbow	5.3	25.0	47	
Right Median Motor (Abductor Pollicis Brevis)											
Wrist	4.1*	4.30	16.46	>7	7.2	Wrist	Wrist	0.0	5.0	-	>50
Elbow	8.5	4.38	16.16		7.1	Wrist	Elbow	4.4	22.0	50	
Left Ulnar Motor (Abductor Digiti Minimi)											
Wrist	2.8	5.70	7.38	>7	2.4*	Wrist	Wrist	0.0	5.0	-	>50
B Elbow	8.7	6.88	5.15		1.5	Wrist	B Elbow	5.9	20.0	34	
A Elbow	10.2	7.19	4.31		1.2	B Elbow	A Elbow	1.5	10.0	67	
Right Ulnar Motor (Abductor Digiti Minimi)											
Wrist	2.3	4.69	19.98	>7	7.8	Wrist	Wrist	0.0	5.0	-	>50
B Elbow	6.3	5.00	19.77		7.6	Wrist	B Elbow	4.0	21.0	53	
A Elbow	8.0	5.00	18.15		6.5	B Elbow	A Elbow	1.7	10.0	59	

**Table 3:** Nerve Conduction F Wave Studies ms, milliseconds; L-R, left-to-right *Abnormal values

F-Latency (ms)	Latency Normal (ms)	L-R F-Latency (ms)	L-R Latency Normal
Left Median (Markers) (Abductor Pollicis Brevis)			
34.96*	<31.1	6.30*	<2.2
Right Median (Markers) (Abductor Pollicis Brevis)			
28.67	<31.1	6.30*	<2.2
Left Ulnar (Markers) (Abductor Digiti Minimi)			
38.30*	<31.7	11.64*	<2.5
Right Ulnar (Markers) (Abductor Digiti Minimi)			
26.67	<31.7	11.64*	<2.5

**Table 4:** Electromyogram 1st Dor Int, first dorsal interosseous; Musculocut, musculocutaneous; Abd Poll Brev, abductor pollicis brevis; Flex Dig Prof, flexor digitorum profundus *Abnormal values

Side	Muscle	Nerve	Root	Insertional Activity	Fibrillation	Positive Sharp Wave	Fasciculations	Amplitude	Duration	Polyphase	Recruitment	Interference Pattern	Comment
Left	1stDorInt	Ulnar	C8-T1	Increased*	3+*	3+*	0	-	-	-	-	-	No activation*
Left	Pronator Teres	Median	C6-7	Increased*	3+*	3+*	0	-	-	-	-	-	No activation*
Left	Biceps	Musculocut	C5-6	Increased*	3+*	3+*	0	-	-	-	-	-	No activation*
Left	Triceps	Radial	C6-7-8	Increased*	2+*	3+*	0	Normal/1+*	1+*	Normal	-1*	-3*	-
Left	Deltoid	Axillary	C5-6	Increased*	3+*	4+*	0	-	-	1+*	-	-3*	-
Right	1stDorInt	Ulnar	C8-T1	Increased*	3+*	3+*	0	1+*	Normal/1+*	Normal	-1*	Normal	-
Right	Abd Poll Brevis	Median	C8-T1	Normal	0	0	0	Normal/1+*	1+*	1+*	-1*	Normal	-
Right	Pronator Teres	Median	C6-7	Normal	0	0	0	Normal	Normal/1+*	1+*	Normal/1-*	Normal	-
Right	Flex Dig Prof	Ulnar	C8,T1	Normal	0	0	0	Normal/1+*	1+*	1+*	-1*	Normal	-
Right	Biceps	Musculocut	C5-6	Normal	0	0	0	Normal/1+*	1+*	1+*	-2*	Normal	-
Right	Triceps	Radial	C6-7-8	Normal	0	0	0	Normal	1+*	Normal	-1*	Normal	-
Right	Deltoid	Axillary	C5-6	Normal	0	0	0	Normal	Normal/1+*	Normal	Normal/1-*	Normal	-

**Figure 1:**
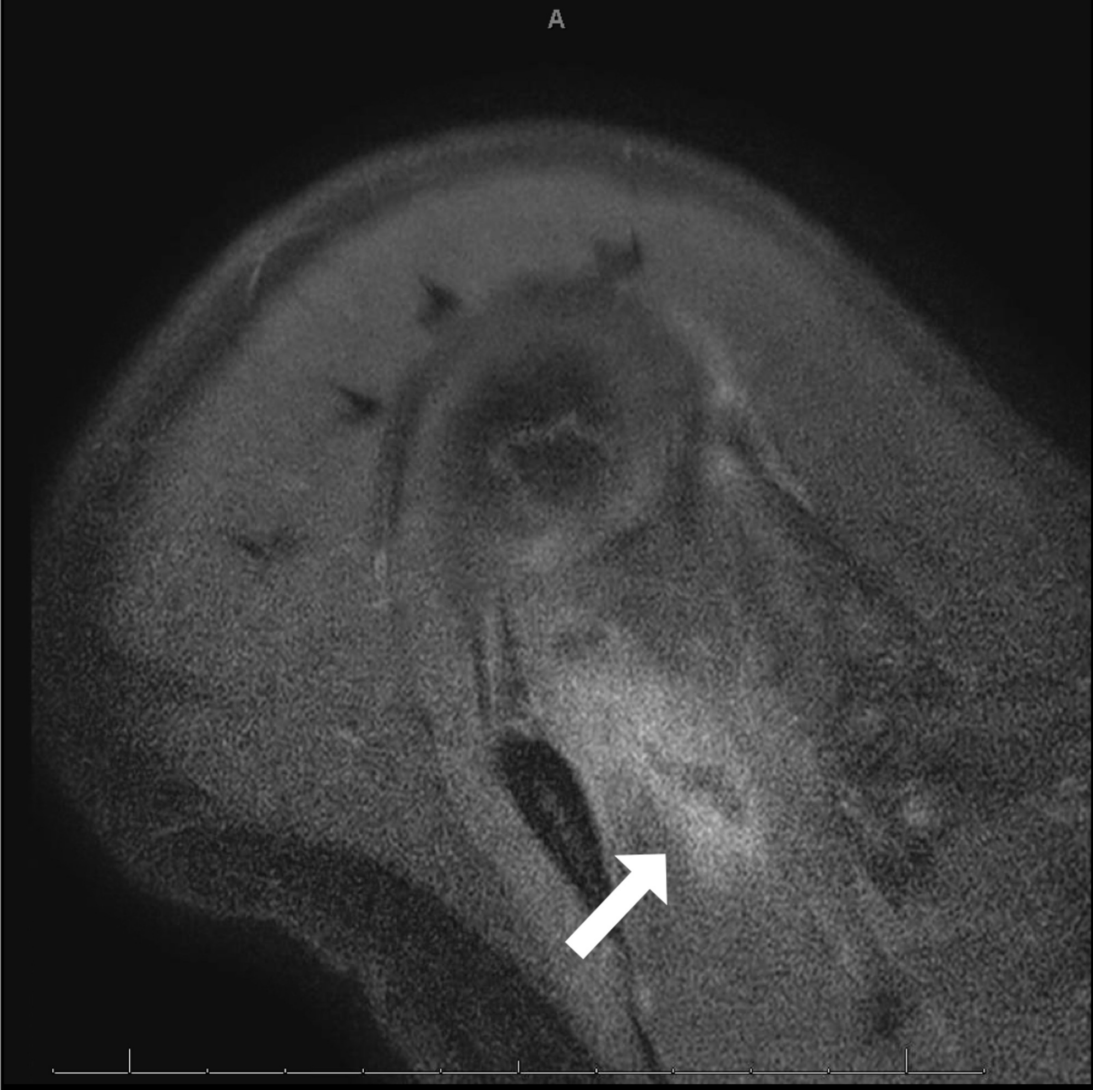
Right axial T1-weighted MRI showing supraspinatus lesion with irregular peripheral edema and non-enhancing central core suggestive of myonecrosis. MRI, magnetic resonance imaging

He underwent digital subtraction magnetic resonance angiography of the left shoulder to evaluate for vascular causes of necrosis, which was unrevealing. Contrast-enhanced computed tomography (CT) of the chest, abdomen, and pelvis to evaluate for occult malignancy was negative. MRI of the cervical spine showed chronic changes without acute abnormalities, which could explain his presentation. MRI of his right shoulder and brachial plexus revealed diffuse edema of the right shoulder and right supraspinatus muscles with a central lesion consistent with myonecrosis, osteonecrosis of the right humeral head, and chronic degenerative changes of the right shoulder joint (Figure 1).

**Figure 2:**
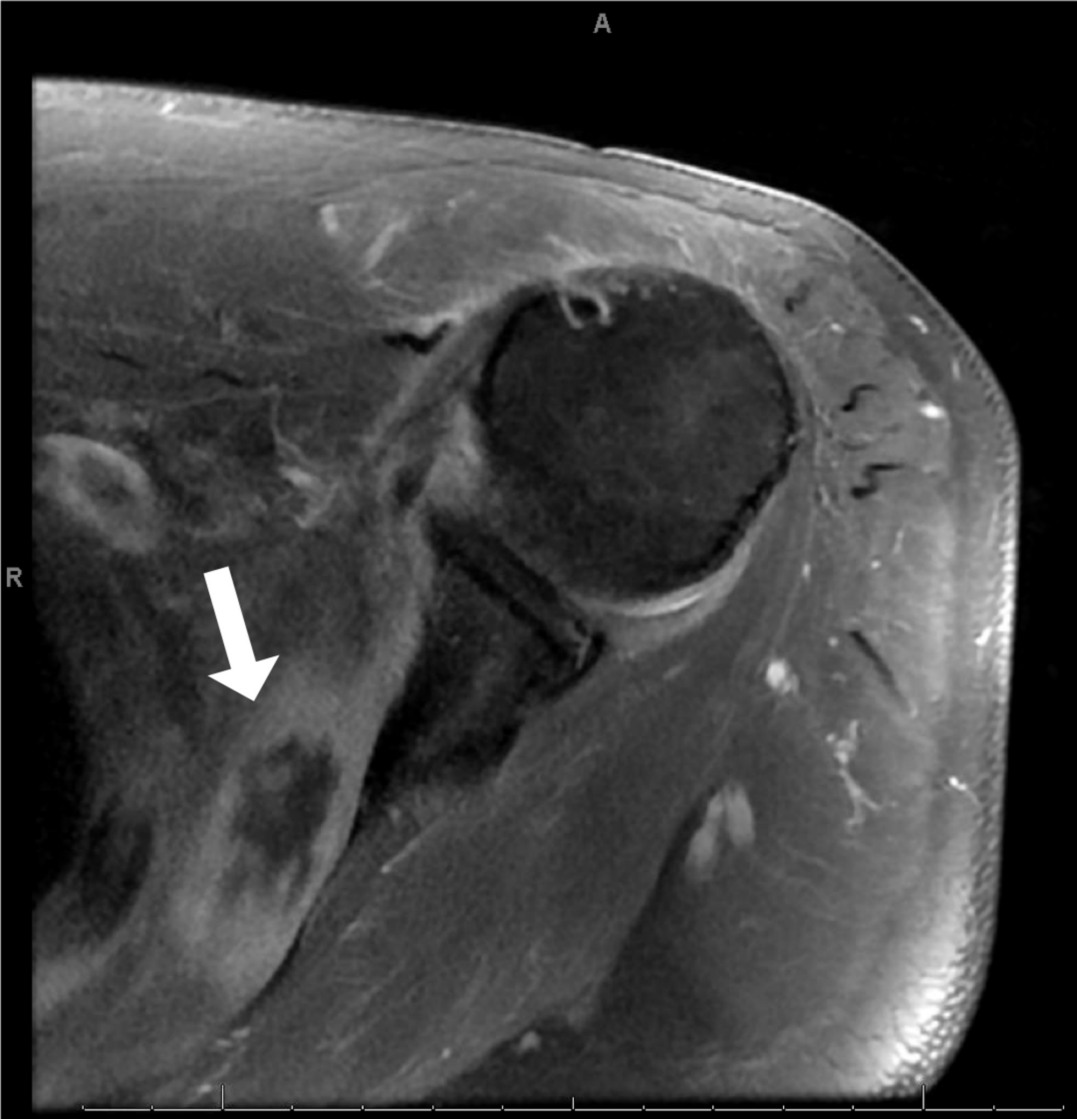
Left axial T1-weighted MRI showing subscapularis lesion with irregular peripheral edema and non-enhancing central core suggestive of myonecrosis. MRI, magnetic resonance imaging

MRI of the left shoulder showed similar findings to the right with myonecrosis of the subscapularis muscle (Figure 2).

Imaging at this point was unable to exclude resolving infectious pyomyositis. Extensive diagnostic evaluation for infectious and rheumatologic etiologies was negative (Table 5). A repeat muscle biopsy from a left deltoid region with radiologic evidence of edema and inflammation was performed to obtain 16s ribosomal RNA PCR (polymerase chain reaction) studies. Results of these additional studies returned negative for microbial pathogens (Table 5).

**Table 5:** Diagnostic laboratory results CSF, cerebrospinal fluid; ANA, anti-nuclear antibody; HLA, human leukocyte antigen; PCR, polymerase chain reaction; rRNA, ribosomal ribonuclear acid; RF Quant, rheumatoid factor quantitative test; IgG, immunoglobulin G; SSA, Sjögren syndrome related antigen A; IgM, immunoglobulin M; WBC, white blood cell; AFB, acid-fast bacillus; SSB, Sjögren’s syndrome related antigen B; VZV, Varicella zoster virus; RBC, red blood cell; c-ANCA, cytoplasmic anti-neutrophil cytoplasmic antibody; p-ANCA, perinuclear anti-neutrophil cytoplasmic antibody; CMV, cytomegalovirus; DNA, deoxyribonucleic acid; Quant, quantitative; MPO, myeloperoxidase; HSV, herpes simplex virus; Ab, antibody; PR3, proteinase 3; EBV, Epstein-Barr virus; HHV-8, human herpesvirus 8; Ag, antigen; VDRL, venereal disease research laboratory test; NA, nuclear antigen; SRP, signal recognition particle; HIV, human immunodeficiency virus

Serum test	Result	Serum Test	Result		CSF Studies	Result	Left Deltoid Biopsy	Result
ANA	(–)	HLA-B27	(–)		Glucose	63 mg/dL	16S rRNA Bacterial PCR	(–)
RF Quant	(–)	West Nile Virus IgG	(–)		Protein	73 mg/dL	16S rRNA Fungal PCR	(–)
Anti-SSA (anti-ro)	(–)	West Nile Virus IgM	(–)		WBC	2 /mm^3^	16S rRNA AFB PCR	(–)
Anti-SSB (anti-la)	(–)	VZV IgG	(+)		RBC	0 /mm^3^	Gram Stain	(–)
c-ANCA	(–)	VZV IgM	(–)		Color	Clear	Bacterial Culture	(–)
p-ANCA	(–)	VZV PCR	(–)		CMV DNA Quant	(–)	VZV PCR	(–)
Anti-MPO	(–)	HSV PCR	(–)		VZV Total Ab	(–)	HSV PCR	(–)
Anti-PR3	(–)	EBV PCR	(–)		VZV IgM	(–)	HHV-8 PCR	(–)
Anti-MI-2	(–)	EBV IgG Capsid	(+)		Cryptococcal Ag	(–)		
Anti-PL-7	(–)	EBV IgM Capsid	(–)		VDRL	(–)		
Anti-PL-12	(–)	EBV NA IgG	(+)		Bacterial Culture	(–)		
Anti-EJ	(–)	CMV PCR	(–)		AFB Culture	(–)		
Anti-OJ	(–)	CMV IgM	(–)		Fungal Culture	(–)		
Anti-SRP	(–)	Parvovirus B19 IgG	(+)		VZV PCR	(–)		
Anti-Jo-1	(–)	Parvovirus B19 IgM	(–)		CMV PCR	(–)		
Anti-KU	(–)	Treponemal Ab	(–)		West Nile Virus PCR	(–)		
Anti-U2 SN RNP	(–)	HIV 1&2 Ab, Ag	(–)		EBV PCR	(–)		
Aldolase	(–)	Bacterial Culture	(–)		HSV PCR	(–)		
Cryoglobulins	(–)	AFB Culture	(–)					

He continued to experience severe pain requiring multiple pain medications with minimal relief of symptoms, and three months following his initial presentation he received an empiric trial of methylprednisolone followed by plasma exchange therapy (PLEX), with neither alleviating his symptoms. At eight months after the initial onset, serial MRIs of both the shoulders have shown mild interval improvement of multifocal edema and myonecrosis, but he remained symptomatic with minimal relief and no definitive etiology of his symptoms identified. He received an additional trial of intravenous immunoglobulin (IVIG), with minimal effect on pain or functional status of his left arm. Having exhausted numerous diagnostic avenues and several trials of therapy with negligible improvement, his treatment goals shifted to comprehensive pain management and physical rehabilitation.

## Discussion

**Figure 3:**
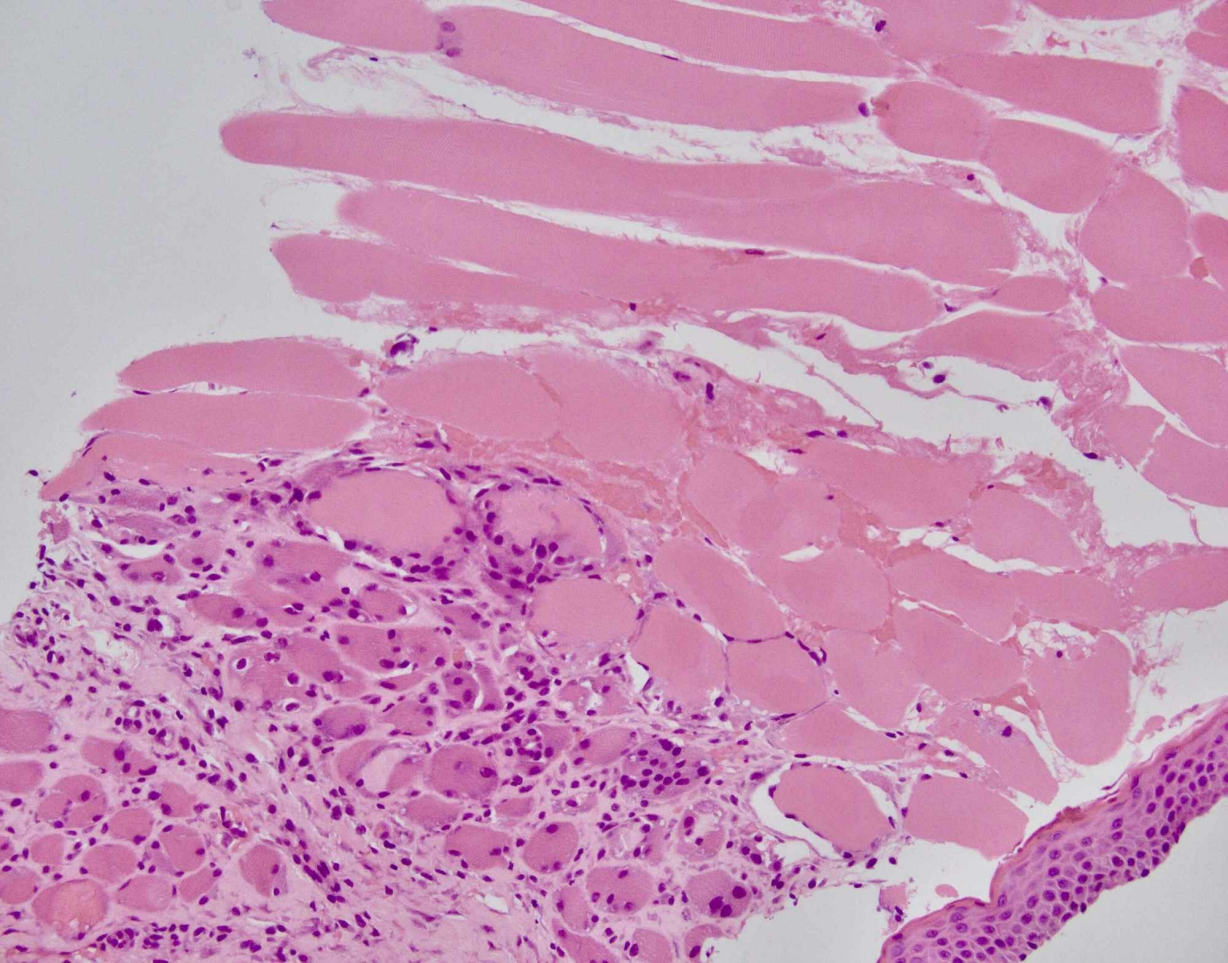
Left subscapularis core biopsy showing atrophic skeletal muscle with chronic inflammation and fibrosis (bottom) progressing to myonecrosis (top).

Manifestations of NA have been well documented to include diffuse edema and inflammation of the shoulder girdle muscles as well as focal muscle atrophy, but this appears to be the first report of a case of NA that progressed to frank necrosis of the involved muscles and rhabdomyolysis [2,3,5-7]. Our patient displayed several classical findings of NA including sudden-onset severe unilateral arm pain that awakened him from sleep, as well as LUE paralysis and scapular winging. However, the myonecrosis seen on imaging presented a diagnostic challenge. Imaging findings in our patient demonstrated signal enhancement in numerous muscles of the shoulder girdle and trunk, as well as the brachial plexus, suggesting edema and inflammation, with irregular foci of edema and non-enhancing central cores consistent with myonecrosis of the right supraspinatus and left subscapularis muscles (Figures 1, 2). Myonecrosis was confirmed with biopsy showing inflammation and necrotic muscle tissue (Figure 3).

The causes of NA are unclear but are thought to be multifactorial, including environmental, mechanical, and genetic factors [5]. As is often the case with this syndrome, we were unable to identify a definitive trigger for NA in our patient. He had no personal or family history of NA, though he had an extensive remote history of trauma and surgeries on his cervical spine related to his military service. A case series by Brown et al. describes the development of NA post-cervical decompression in a series of six patients, though, unlike our case, these patients experienced onset within hours to days of their surgeries [9]. Given reports of biomechanical factors implicated in the pathophysiology of NA, our patient’s occupational trauma, while remote, was also considered as a potential predisposing factor to his presentation [1,5]. Our patient’s military service involved frequent deep-sea dives and high-altitude jumps, resulting in dysbaric osteonecrosis of both hips, and our imaging uncovered additional evidence of osteonecrosis of his right humeral head, suggesting a significant cumulative barotrauma burden. Digital subtraction angiogram was negative for macrovascular ischemia, but it is unclear whether the same chronic gaseous emboli that resulted in his dysbaric osteonecrosis may have also contributed to chronic microvascular insufficiency in the nerves and soft tissues of his shoulder girdle, predisposing to the development of NA.

Given his atypical presentation with multifocal myonecrosis and rhabdomyolysis suggesting a possible infectious etiology, a subclinical infectious illness prior to presentation was considered. His travel while serving was remote but included countries ranging from Central America and Africa to the Middle East and Asia, raising the possibility of unusual exposures such as fungal and atypical mycobacterial infections. Much of the literature describing infectious triggers for NA consists of case reports, but one large case series of 246 patients with NA estimates that up to 43% of idiopathic NA may be associated with an antecedent infection [3]. Several infections have been implicated, many of which are common, including Epstein Barr virus (EBV), Varicella zoster virus (VZV), herpes simplex virus (HSV), cytomegalovirus (CMV), parvovirus B19, hepatitis E virus, Mycoplasma pneumoniae, and Lyme disease. Many cases of NA have been reported as a manifestation of infectious mononucleosis with documented evidence of EBV infection. CMV and parvovirus infections have likewise been implicated. Patients with HSV or VZV triggering NA typically have classic lesions of the mouth or skin. Patients reported to have hepatitis E virus triggering NA usually present with fulminant viral hepatitis, but they may also present with more subacute cholestatic liver disease. There have also been reports of unilateral brachial plexus neuropathy in a patient with mycoplasma pneumoniae causing lobar pneumonia. Our patient did not present with features suggesting a specific infectious etiology, though some patients without a clear history of infection may yield evidence of infectious triggers with additional diagnostic workup. For example, patients with NA have been reported to have serum or cerebrospinal fluid serologies test positive for Borrelia burgdorferi despite lacking clinical symptoms or history of Lyme disease. Treatment for neurologic Lyme disease in these patients has resulted in improvement in symptoms. We pursued broad diagnostic testing for infections that could trigger NA in our patient; however, all serologic and nucleic acid amplification tests performed on his serum, cerebrospinal fluid, and muscle tissue were negative for microbial pathogens (Table 5). We also considered other causes of myopathy such as sarcoidosis; however, our patient lacked fever, rash, arthralgias, dyspnea, or other clinical manifestations or imaging findings typical of sarcoidosis. Furthermore, none of the tissue samples showed evidence of non-caseating granulomas.

There have been no published controlled trials of therapy in NA [10]. As the presumed mechanism of pathogenesis is immune-mediated, most therapies center on immunomodulation such as corticosteroids, IVIG, and PLEX. However, the existing literature is limited and consists only of retrospective and anecdotal evidence [10-20]. van Eijk et al. report a controlled retrospective study of 50 patients with NA treated with prednisolone in the acute phase (within 31 days). Compared with historical controls, treated patients had significantly lower median time to pain relief, higher percentage of strength recovery in the first month of treatment, and full recovery at one year [20]. van Alfen and van Engelen report 41 patients who were treated with corticosteroids [3]. Compared with controls, the only significant difference in treated patients was shorter time to start paresis recovery. The authors detected no difference in the duration of initial pain, maximum pain score, chronic pain, or strength recovery. Naito et al. report a case series of 15 patients with NA, 10 of whom received combination IVIG and methylprednisolone. The median interval between symptom onset and treatment was three months, and all 10 patients showed improvement of motor dysfunction within one month after initiation of therapy [14]. Nakajima et al. and Ardolino et al. report cases of NA that showed dramatic improvement in pain and motor function within days of initiating treatment with IVIG [13,17]. Several additional cases of patients treated successfully with IVIG have been reported, but it is unclear if these cases represent a therapeutic benefit of IVIG or simply the natural history of NA [5,16,18]. Physical therapy has also been proposed as a treatment modality given the disabling nature of NA, but evidence for its benefit is also minimal [12]. It would seem that the treatments for NA with the strongest evidence are IVIG and corticosteroids, but these must be instituted early to confer benefit [10,11,20]. Though our patient received courses of corticosteroids, PLEX, and IVIG, he was initiated several months after onset of symptoms and did not produce significant clinical improvement. While his initial symptoms fit well with the classic findings of NA, the atypical presentation with myonecrosis delayed both the definitive diagnosis and the initiation of immunomodulating therapy.

Pain in NA is usually refractory to acetaminophen or non-steroidal anti-inflammatory drugs (NSAIDs) alone, but van Alfen and van Engelen showed that the most effective therapy was an opioid analgesic combined with an NSAID, which was effective in 60% of patients [3]. Recovery occurs at times within months but can take up to 3-8 years, and 20-66% of patients can experience persistent, long-term pain, weakness, and disability. Persistent symptoms at three months without improvement is a poor prognostic sign [1,2,4].

## Conclusions

While the majority of patients with NA will present classically, there is a significant minority of patients who will present with atypical findings. This is the first report of a case of NA presenting with multifocal myonecrosis of involved muscles and rhabdomyolysis. Such an unusual presentation, if unrecognized, can result in diagnostic and therapeutic delays. It is important to recognize this manifestation of NA in order to ensure a timely initiation of immunomodulating therapy, comprehensive pain management, and rehabilitation to maximize functional capacity and minimize morbidity.
